# The oncogene *KRAS* promotes cancer cell dissemination by stabilizing spheroid formation via the MEK pathway

**DOI:** 10.1186/s12885-018-4922-4

**Published:** 2018-12-03

**Authors:** Juri Ogishima, Ayumi Taguchi, Akira Kawata, Kei Kawana, Mitsuyo Yoshida, Yuki Yoshimatsu, Masakazu Sato, Hiroe Nakamura, Yoshiko Kawata, Akira Nishijima, Asaha Fujimoto, Kensuke Tomio, Katsuyuki Adachi, Takeshi Nagamatsu, Katsutoshi Oda, Tohru Kiyono, Yutaka Osuga, Tomoyuki Fujii

**Affiliations:** 1 Department of Obstetrics and Gynecology, Graduate School of Medicine, The University of Tokyo, 7-3-1 Hongo, Bunkyo-ku, Tokyo, 113-8655 Japan; 2 Department of Obstetrics and Gynecology, Graduate School of Medicine, Nihon University, 30-1 Otaniguchi Uemachi, Itabashi-ku, Tokyo, 173-8610 Japan; 3 Division of Carcinogenesis and Cancer Prevention, National Cancer Center Research Institute, 5-1-1 Tsukiji, Chuo-ku, Tokyo, 104-0045 Japan

**Keywords:** Spheroid formation, KRAS, MEK pathway, MEK inhibitor, Trametinib

## Abstract

**Background:**

Peritoneal dissemination is a critical prognostic factor in ovarian cancer. Although stabilized spheroid formation promotes cancer cell peritoneal dissemination in ovarian cancer, the associated oncogenes are unknown. In this study, we assessed the role of the *KRAS* oncogene in ovarian cancer cell dissemination, focusing on the stability of cells in spheroid condition, as well as the modulation of intracellular signaling following spheroid transformation.

**Methods:**

We used ID8, a murine ovarian cancer cell line, and ID8-KRAS, an oncogenic KRAS (G12 V)-transduced ID8 cell line in this study. Spheroid-forming (3D) culture and cell proliferation assays were performed to evaluate the growth characteristics of these cells. cDNA microarray analysis was performed to identify genes involved in KRAS-associated signal transduction in floating condition. A MEK inhibitor was used to evaluate the effect on cancer peritoneal dissemination.

**Results:**

Cell viability and proliferation in monolayer (2D) cultures did not differ between ID8 and ID8-KRAS cells. However, the proportions of viable and proliferating ID8-KRAS cells in 3D culture were approximately 2-fold and 5-fold higher than that of ID8, respectively. Spheroid-formation was increased in ID8-KRAS cells. Analysis of peritoneal floating cells obtained from mice intra-peritoneally injected with cancer cells revealed that the proportion of proliferating cancer cells was approximately 2-fold higher with ID8-KRAS than with ID8 cells. Comprehensive cDNA microarray analysis revealed that pathways related to cell proliferation, and cell cycle checkpoint and regulation were upregulated specifically in ID8-KRAS cells in 3D culture, and that some genes partially regulated by the MEK-ERK pathway were upregulated only in ID8-KRAS cells in 3D culture. Furthermore, a MEK inhibitor, trametinib, suppressed spheroid formation in 3D culture of ID8-KRAS cells, although trametinib did not affect 2D-culture cell proliferation. Finally, we demonstrated that trametinib dramatically improved the prognosis for mice with ID8-KRAS tumors in an in vivo mouse model.

**Conclusions:**

Our data indicated that KRAS promoted ovarian cancer dissemination by stabilizing spheroid formation and that the MEK pathway is important for stabilized spheroid formation. Disruption of spheroid formation by a MEK inhibitor could be a therapeutic target for cancer peritoneal dissemination.

**Electronic supplementary material:**

The online version of this article (10.1186/s12885-018-4922-4) contains supplementary material, which is available to authorized users.

## Background

Although platinum-based anti-cancer therapy has a high response rate for ovarian cancer, the five-year survival rate of patients with peritoneally disseminated advanced ovarian cancer remains less than 40% [1]. A novel platinum-based regimen combined with molecular-targeting agents, including bevacizumab or a poly (ADP-ribose) polymerase inhibitor, does not improve the overall survival for all patients with advanced ovarian cancer [2, 3]. Advanced ovarian cancer with peritoneal dissemination to the upper abdomen (pT3) is difficult to control using conventional chemotherapy, including molecular-targeting agents. The peritoneal recurrence rate after primary standard therapy is approximately 80% [1].

The relationship between ovarian cancer and oncogenes has been extensively studied. Type 1 ovarian cancers, which consist of low-grade serous carcinoma (LGSC), mucinous carcinoma, endometrioid carcinoma, malignant Brenner tumor, and clear cell carcinoma typically display a variety of somatic sequence mutations in *KRAS*, *BRAF*, *PTEN*, *PIK3CA CTNNB1*, *ARID1A*, and *PPPWR1A*, but very rarely in *TP53* [4–6]. In particular, approximately 40% of type 1 ovarian cancers have *KRAS* mutation (https://www.mycancergenome.org/content/disease/ovarian-cancer/kras). In addition, a previous report demonstrated that metastatic type 1 ovarian cancer with *KRAS* mutation has poor prognosis compared to those without it [7]. In contrast, type 2 tumors, which consist of high-grade serous carcinoma (HGSC), high-grade endometrioid carcinoma, malignant mixed mesodermal tumors, and undifferentiated carcinomas, are chromosomally highly unstable and harbor *TP53* mutations in > 95% cases [8]. The Cancer Genome Atlas (TCGA) research network reported that amplification of *KRAS* is observed in at least 10% of HGSCs. It also reported that patients with wild-type *TP53* had significantly shorter survival and higher chemoresistance than those with mutated *TP53* [9]. Importantly, one-third of patients with wild-type *TP53* harbor either *RAS* mutations or activated mutations of RAS-related molecules. *KRAS* mutation is also observed in borderline tumors, and patients of serous borderline tumors with KRAS G12 V mutation have shorter survival time than those without it [10]. Similarly, the oncogene *KRAS* plays an important role in ovarian cancer.

The mechanisms of carcinogenesis of mutated oncogenes are well-studied. In ovarian, tubal, and peritoneal cancers, the oncogenes initiate immortalization, enhance cell proliferation, and inhibit cancer cell apoptosis [11–14]. These changes accompany tumor formation, sometimes followed by dissemination of the cancer cells in the peritoneum. The tumor formation and dissemination microenvironments are considerably different, and the additional assistance provided by oncogenes is a prerequisite for peritoneal dissemination. In our previous study, we compared the mouse ovarian cancer cell lines ID8 and ID8-KRAS (oncogenic KRAS (G12 V) transduced in the ID8 cell line) and observed that KRAS promoted rapid tumor formation with severe inflammation in vivo mouse models, although in vitro, ID8 cell proliferation was not affected by KRAS [15]. Our previous results suggested that KRAS accelerates tumor formation by modulating the tumor microenvironment (TME) and favoring peritoneal dissemination through production of granulocyte macrophage colony-stimulating factor, followed by neutrophil infiltration [15].

The most widely described mechanism for peritoneal dissemination is that cancer cells detach from the primary tumor, spread to the peritoneal cavity via the peritoneal fluid, and attach and seed in the peritoneum [11]. Several previous studies have shown that peritoneal dissemination of ovarian cancer is associated with the spheroid-forming capacity of cancer cells [16–18]. This suggests that floating cells derived from primary tumors can disseminate to the peritoneum if the cancer cells proliferate stably under 3D or floating conditions.

Here, we hypothesized that differences in tumor formation between ID8 and ID8-KRAS cells might be caused not only by TME modulation, but also by spheroid formation (anchorage-independent growth). Several previous studies have shown that the *KRAS* oncogene contributes to anchorage independence of cancer cells. In non-small cell lung cancer (NSCLC) with *KRAS* mutation, *KRAS* knock down did not affect cell proliferation in the 2D culture condition, whereas it markedly decreased soft-agar colony formation, suggesting that KRAS is important for anchorage-independent growth of NSCLC cells [19]. Others have demonstrated that mitochondrial metabolism and mitochondrial reactive oxygen species generation are essential for KRAS-induced cell proliferation and tumorigenesis [20]. Similarly, KRAS is expected to be closely related to anchorage-independent cancer cell growth in colorectal cancer cell lines [21]. In this study, we assessed the role of the *KRAS* oncogene in ovarian cancer cell dissemination, focusing on the stability of cells in spheroid condition, as well as the modulation of intracellular signaling following spheroid transformation.

## Methods

### Cell lines

The mouse ovarian cancer cell line ID8 was a kind gift from Dr. Kathy Roby, Department of Anatomy and Cell Biology, University of Kansas Medical Center. An oncogenic mutant form of human KRAS (KRASG12 V) was recombined into pDEST-CLXSN to generate pCLXSN-KRASG12V. Retrovirus packaging was performed as previously described [22]. ID8-KRAS cells were established by infection of the LXSN-KRASG12 V virus at multiplicity of infection of 1, followed by G418 selection at a concentration of 800 μg/ml for 1 week [15]. By western blotting, pMEK1/2 seemed to be increased in ID8-KRAS cells compared to ID8 cells, however there was no significant difference between two cell lines (*p* = 0.92) (Additional file 1: Supplementary Methods and Additional file 2: Figure S1). The ID8 and ID8-KRAS cells were transduced with GFP-expressing lentiviral particles to produce ID8-GFP and ID8-KRAS-GFP cells.

ID8-GFP and ID8-KRAS-GFP cells were cultured in Dulbecco’s modified Eagle’s medium (DMEM) (Wako Pure Chemical Industries, Ltd. (Wako), Osaka, Japan) containing 10% fetal bovine serum (FBS; Invitrogen, Carlsbad, CA, USA), 100 U/ml penicillin, 0.1 mg/ml streptomycin (Wako). All cell lines were incubated at 37 °C in the presence of 5% CO_2_.

### Mouse model

C57BL/6 J mice were used because ID8 was established from this strain of mice [23]. Mice were purchased from Japan SLC, Inc. (Shizuoka, Japan). ID8-GFP and ID8-KRAS-GFP cells (1 × 10^6^) suspended in 1000 μl DMEM were injected into the peritoneal cavities of 8-week-old female mice, the average body weight (BW) of which was 18 g, under isoflurane anesthesia [15]. Mice were housed under specific pathogen-free conditions and 12 h light/dark cycle in metal cages, with five mice per cage. The bedding material was wooden. Mice were sacrificed by isoflurane overdose, and sacrificed to minimize suffering when moribund behaviors were observed. For the mice survival analysis, mice were sacrificed when their BW exceeded 23 g after cell line inoculation, because in our previous study [15], we confirmed that approximately 5 ml of ascites accumulated with tumor formation when BW reached 23 g. BW and ascites weight were assessed at the time of sacrifice. The total number of mice used in this study was 44, and the mice were randomly divided as follows for various experiments: for cell proliferation assay, six each of ID8 and ID8-KRAS mice were used; for apoptosis analysis, six each of ID8 and ID8-KRAS mice were used; for treatment with the MEK inhibitor, ten each of treated and control mice were used.

### Spheroid-forming (3D) culture

Dissociated single cells (1 × 10^5^ cells/ml) were seeded into ultra-low attachment plates (Corning, USA) and were cultured for 48 h [24]. Ten-centimeter dishes were used for microarray analysis, cell counting, and cell proliferation assays, whereas 6-well plates were used for MEK inhibitor treatment. For collecting spheroids, the medium was centrifuged for 2 min at 100×*g* and dissociated into single cells using 0.25% trypsin/EDTA (Wako).

### In vitro cell proliferation assay

On the first day, ID8-GFP and ID8-KRAS-GFP cells (1 × 10^6^) were plated onto a 10-cm dish in serum-containing media and cultured for 48 h, following which, the cells were collected, and live, trypan blue-excluding cells were counted to determine the number of viable cells.

For proliferation analysis using 5-ethynyl-2′-deoxyuridine (EdU), the cells were exposed to EdU (10 μM) for 2 h on the third day after 48 h of seeding ID8-GFP or ID8-KRAS-GFP cells [25]. After 2 h of EdU administration, attached and floating cells in a 10-cm dish were collected and washed with phosphate buffered saline (PBS). To detect EdU incorporation, EdU was labeled with Alexa Fluor 647 using the Click-iT Plus EdU Alexa Fluor 647 flow cytometry assay kit (Life Technologies, MA, USA). EdU-stained cells were fixed according to the manufacturer’s protocol, and they were run on a FACSCalibur flow cytometer (BD Biosciences, NJ, USA). All cells were collected and gated based on the GFP-positive area. The data were analyzed using FlowLogic software (Inivai Technologies, Melbourne, Australia). The proportion of proliferating cells was calculated from the following formula.

$$ \mathrm{The}\ \mathrm{proportion}\ \mathrm{of}\ \mathrm{proliferating}\ \mathrm{cells}\ \left(\%\right)=100\times \left(\mathrm{Number}\ \mathrm{of}\ \mathrm{EdU}-\mathrm{positive}\ \mathrm{cells}\ \mathrm{in}\ \mathrm{GFP}-\mathrm{positive}\ \mathrm{area}\right)/\left(\mathrm{Number}\ \mathrm{of}\ \mathrm{GFP}-\mathrm{positive}\ \mathrm{cells}\right) $$

### In vivo cell proliferation assay

On the first day, mice were injected via the intraperitoneal route (i.p.) with 1 × 10^6^ ID8-GFP or ID8-KRAS-GFP cells. Two days after injection of ID8-GFP or ID8-KRAS-GFP cells, mice were i.p. injected with 40 μl EdU (200 μg, 20 mM) in PBS (500 μl) [26, 27]. After 2 h of EdU administration, 8 ml of normal saline was i.p. injected into mice, and cells were recovered from the peritoneal cavity using peritoneal washes and passed through a cell strainer. Approximately 1000,000 cells were obtained routinely from the recovered peritoneal washes per mouse. The peritoneal washes were centrifuged for 5 min at 1500 rpm, the supernatant was aspirated, and the red blood cells were lysed. To detect EdU incorporation, EdU was labeled with Alexa Fluor 647 using the Click-iT Plus EdU Alexa Fluor 647 flow cytometry assay kit (Life Technologies) per manufacturer’s protocol. EdU-stained cells were fixed according to the manufacturer’s protocol, and they were run on a FACSCalibur flow cytometer (BD Biosciences). The cancer cell area was recognized by the forward scatter (FSC) versus side scatter (SSC) plot and GFP fluorescence. The data were analyzed using the FlowLogic software (Inivai Technologies). The proportion of proliferating cells was calculated from the following formula.

$$ \mathrm{The}\ \mathrm{proportion}\ \mathrm{of}\ \mathrm{proliferating}\ \mathrm{cell}\mathrm{s}\ \left(\%\right)=100\times \left(\mathrm{Number}\ \mathrm{of}\ \mathrm{EdU}-\mathrm{positive}\ \mathrm{cell}\mathrm{s}\ \mathrm{in}\ \mathrm{the}\ \mathrm{cancer}\ \mathrm{cell}\ \mathrm{area}\right)/\left(\mathrm{Number}\ \mathrm{of}\ \mathrm{all}\ \mathrm{cells}\ \mathrm{in}\ \mathrm{the}\ \mathrm{cancer}\ \mathrm{cell}\ \mathrm{area}\right) $$

### Microarray experimental design and data analysis

Total RNA from ID8 and ID8-KRAS cells was extracted using an RNeasy mini kit (QIAGEN, Hilden, Germany). For oligo DNA microarray analysis, RNA samples were collected, and the 3D-Gene mouse oligo chip 24 k (Toray Industries Inc., Tokyo, Japan) was used. For efficient hybridization, this 3D microarray was constructed with a well as the space between the probes and cylinder-stems and 70-mer oligonucleotide probes on the top. Total RNA was labeled with Cy5 using the Amino Allyl MessageAMP II aRNA amplification kit (Applied Biosystems, CA, USA). The Cy5-labeled amino allyl RNA pools and hybridization buffer were hybridized for 16 h. Hybridization was performed using the supplier’s protocols. The hybridization signals were obtained using 3D gene scanner (Toray Industries Inc., Tokyo, Japan), and processed with 3D gene extraction (Toray Industries Inc.). Detected signals for each gene were normalized using the global normalization method (the median of the detected signal intensity was adjusted to 25). Transcripts with a fold change > 2 and *p* values < 0.05 were considered differentially expressed.

### Kyoto encyclopedia of genes and genomes (KEGG) pathway analysis

To extract interpretable information from the differentially expressed gene lists, the microarray data were further investigated using the GeneCodis bioinformatic analysis tool [28, 29]. GeneCodis analyzes functional information by considering the relationships among annotations, such as those generated by KEGG: http://www.genome.jp/kegg/ that are associated with common genes in the list. The GeneCodis analysis was applied to a hypergeometric test, followed by permutation-based correction as a statistical test to determine the annotations that were significantly enriched in a differentially expressed gene list with respect to a reference list of bovine whole transcriptomes in the public database [28–30].

### Reverse transcription-quantitative polymerase chain reaction (RT-qPCR)

RT-qPCR was performed as previously reported [31]. Total RNA was extracted from cultured ID8-GFP and ID8-KRAS-GFP cells using a Favorgen extraction RNA kit (Tokyo, Japan, Chiyoda Science Co.), followed by reverse transcription. cDNA was amplified for 40 cycles in a Light Cycler 480 (Roche, Basel, Switzerland) using SYBR green I (Applied Biosystems). The primer pairs used were as follows: mouse peptidylprolyl isomerase A (PPIA), 5`-CGCGTCTC CTTCGAGCTGTTTG-3′ and 5`-TGTAAAGTCACCACCCTGGCACAT-3′; mouse tescalcin (Tesc), 5`-CCTACCATTCGCAAGGAGAA-3′ and 5`-TTCTCGATGTGAGGGTTTCC-3′; mouse interferon-induced transmembrane protein 1 (IFITM1), 5`-CTTCAAAAGCCGAGAGATG-3′ and 5`-CCACCATCTTCCTGTCCCTA-3′; mouse small proline-rich protein 2A2 (Sprr2a2), 5`-GGTCACTGCTGTTTCATTTCCT-3′ and 5`-ATTAGACCATCACCAAAGGGG-3′; mouse growth-arrest-specific protein 6 (Gas6), 5`-AGGTCTGCCACAACAAACCA-3′ and 5`-GCGTAGTCTAATCACGGGGG-3′; mouse dual-specificity phosphatase 5 (DUSP5), 5`-TGCACCACCCACCTACACTA-3′ and 5`-ATGTCAGCAGTGTGGCTGTC-3′. The PCR conditions used for PPIA were as follows: 35 cycles at 95 °C for 10 s, 63 °C for 10 s and 72 °C for 12 s; for mouse tescalcin, 35 cycles at 95 °C for 10 s, 60 °C for 10 s and 72 °C for 12 s; for mouse IFITM1, 40 cycles at 95 °C for 10 s, 59 °C for 10 s and 72 °C for 7 s; for mouse Sprr2a2, 35 cycles at 95 °C for 10 s, 60 °C for 10 s and 72 °C for 12 s; for mouse Gas6, 40 cycles at 95 °C for 10 s, 63 °C for 10 s and 72 °C for 15 s; and for mouse DUSP5, 35 cycles at 95 °C for 10 s, 63 °C for 10 s and 72 °C for 12 s. The expression of tescalcin, IFITM1, Sprr2a2, Gas6 and DUSP5 was normalized using PPIA mRNA as the internal standard.

### Treatment with MEK inhibitor

For in vitro studies, ID8-KRAS-GFP cells were seeded on 6-well plates in medium containing 10% FBS and immediately treated with the indicated concentration of the MEK inhibitor, trametinib (GSK1120212) (Selleckchem, Houston, TX, USA), or the same volume of dimethyl sulfoxide (DMSO). These cells were incubated for 48 h (see below for additional information for each experimental procedure).

For in vivo studies, treatment started at the time of ID8-KRAS-GFP cell injection. Mice were injected with the MEK inhibitor trametinib (1 mg/kg/day, intraperitoneal injection) or vehicle (0.5% methylcellulose, 0.2% Tween 80, i.p. injection) once per day for 6 days a week for 3 weeks [32]. BW was observed every other day. For the assessment of spheroid formation, mice were sacrificed when BW exceeded 23 g for the control group or on day 35 after ID8-KRAS-GFP cell injection for the trametinib group. Eight milliliters of normal saline was i.p. injected into the sacrificed mice, recovered from peritoneal cavity as peritoneal washes, and passed through a 100 μm cell strainer. The peritoneal washes were centrifuged for 5 min at 1500 rpm the supernatant was aspirated, and the red blood cells were lysed. The buffy coat was aspirated using a dropper, seeded into a 10-cm dish, and spheroid formation was microscopically observed.

### Statistical analysis

Data are presented as means ± standard error of mean (SEM). Statistical analyses were conducted using the Student’s t-test. For multiple comparisons, the *p*-values were adjusted by the Holm’s method for Fig. 4d by using the Microsoft Office Excel 2011 (BellCurve, Tokyo, Japan), and the *p*-values were calculated by the Dunnett’s test for Fig. 5c by using the JMP Pro 13 (SAS Institute Inc., NC, USA). Other statistical analyses were conducted using JMP Pro 13 (SAS Institute Inc.). A value of *p* < 0.05 was considered significant. Asterisks indicate comparisons that were significantly different (p < 0.05). The p-value in microarray analysis was corrected for multiple hypotheses testing using the false discovery rate (FDR) method. Survival was assessed using the log-rank test of the Kaplan-Meier method.

## Results

### KRAS did not promote proliferation in 2D culture but stabilized spheroid formation in 3D culture

**Fig. 1:**
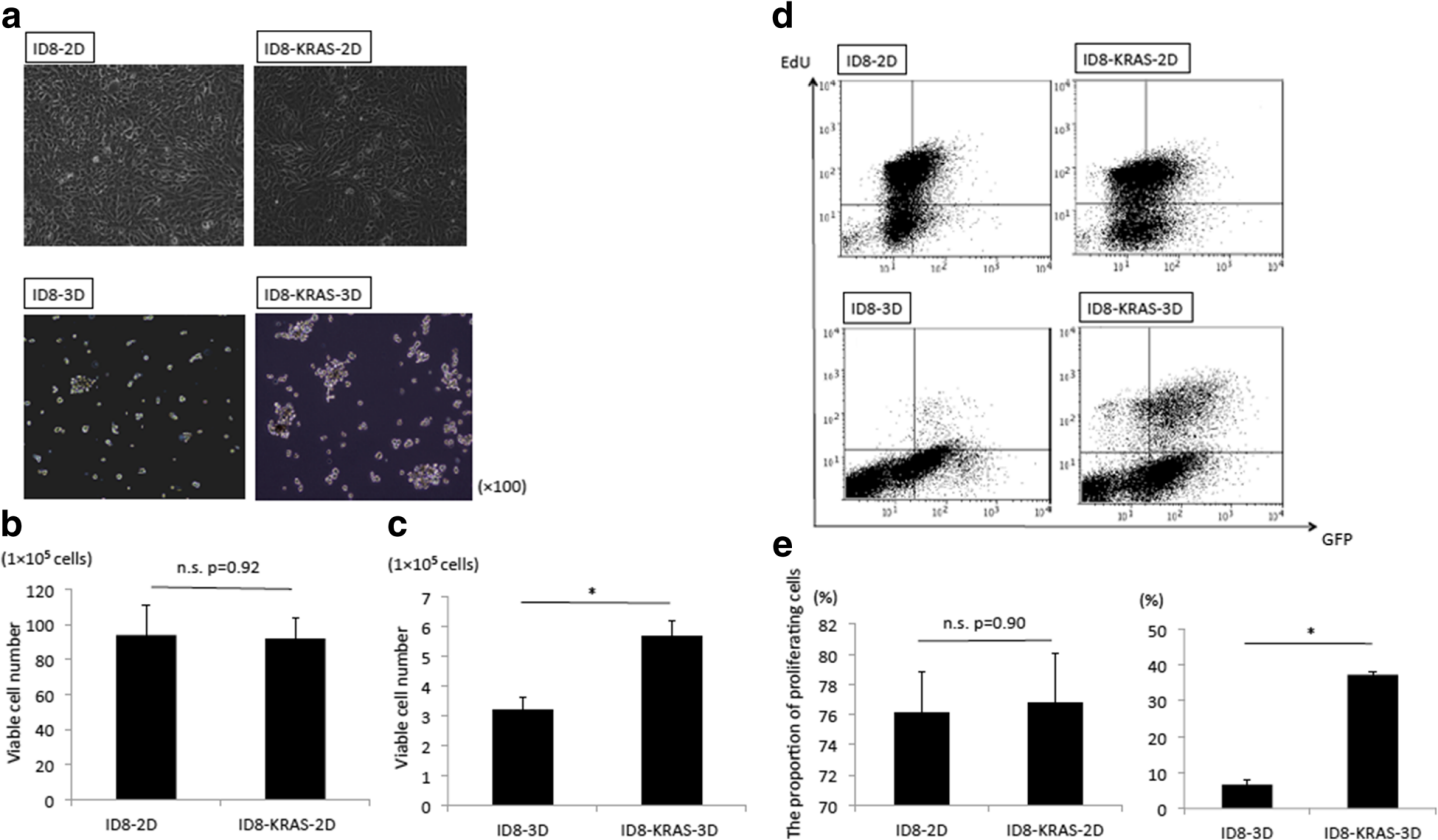
ID8 and ID8-KRAS cells in 2D and 3D conditions in vitro. ID8 and ID8-KRAS cells (1 × 10^6^) were incubated for 48 h in 2D or 3D culture. **a** Representative microscopic images of cancer cells in the 2D and 3D culture conditions at 100 × magnification. b-c Number of viable cells for ID8 and ID8-KRAS in the 2D (**b**) and 3D conditions (**c**). Cultured cells were collected, and live, trypan blue-excluding cells were counted to determine the number of viable cells. Experiments were repeated at least thrice. The values shown represent the mean ± SEM (* *p* < 0.05). d-e ID8-GFP and ID8-KRAS-GFP cells (1 × 10^6^) were incubated for 48 h in 2D or 3D culture. For 5-ethynyl-2′-deoxyuridine (EdU) proliferation analysis, cells were exposed to 10 μM EdU for 2 h before fixation. EdU was labeled with Alexa Fluor 647. EdU uptake in four conditions (ID8-2D, ID8-3D, ID8-KRAS-2D, and ID8-KRAS-3D) was analyzed by flow cytometry (**d**). The proportions of EdU-positive and GFP-positive cells were measured by flow cytometry (**e**). Experiments were repeated at least thrice. The values shown represent the mean ± SEM (* *p* < 0.05)

We compared spheroid formation rates for the ID8 and ID8-KRAS cell lines. We cultured each cell line on a low attachment plate and assessed spheroid formation to mimic the floating condition in the abdomen. There was a marked difference between ID8 and ID8-KRAS cells in 3D culture. In the 3D condition, ID8-KRAS cells formed spheroid structures immediately after incubation and maintained them for 48 h. However, ID8 cells formed few spheroid structures after 48 h incubation (Fig. 1a). The number of viable cells was significantly reduced in both ID8 and ID8-KRAS cells in 3D culture compared to in 2D culture. The decrease was most significant in ID8-3D cultures (ID8-2D (93.9 ± 17.1) × 10^5^cells vs. ID8-KRAS-2D (91.8 ± 11.6) × 10^5^cells, *p* = 0.92, and ID8-3D (3.1 ± 0.4) × 10^5^cells vs. ID8-KRAS-3D (5.6 ± 0.4) × 10^5^cells, *p* < 0.05) (Fig. 1b and c). Next, we assessed EdU (5-ethynyl-2′-deoxyuridine) uptake under ID8-2D, ID8-3D, ID8-KRAS-2D, and ID8-KRAS-3D conditions to evaluate proliferation of each cell type. There was no difference in EdU uptake between ID8-2D and ID8-KRAS-2D cells (76.2 ± 2.7% vs. 76.7 ± 3.3%, *p* = 0.90) (Fig. 1d and e). In contrast, EdU uptake by ID8-3D cells was about 5-fold lower than that of ID8-KRAS-3D cells (6.7 ± 1.5% vs. 37.3 ± 0.8%, p < 0.05) (Fig. 1d and e).

### Differences in expression profiles between ID8 and ID8-KRAS cells under 3D condition

**Fig. 2:**
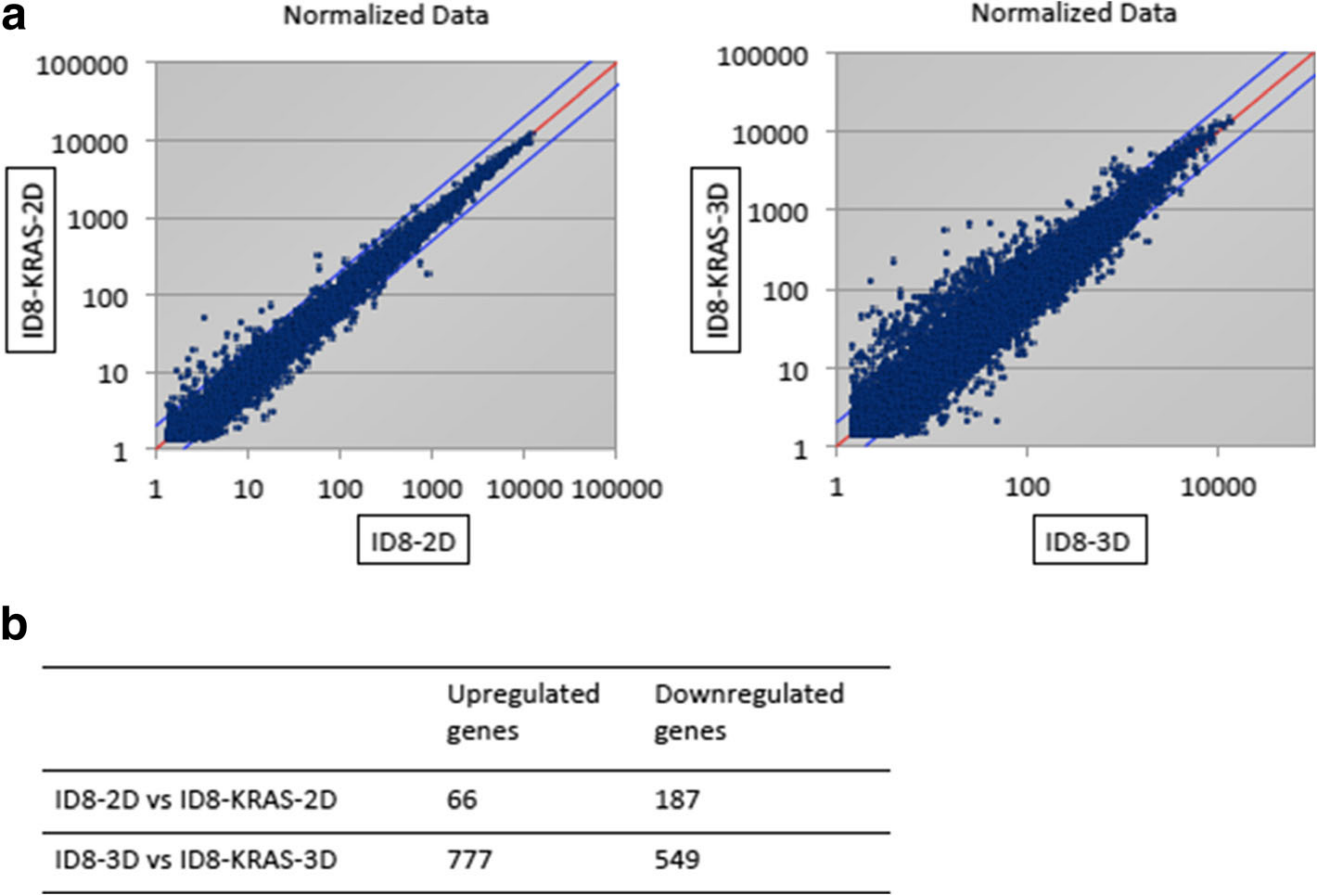
Comparison of cDNA microarray data of ID8-2D and ID8-KRAS-2D with ID8-3D and ID8-KRAS-3D cells, respectively. ID8 and ID8-KRAS cells (1 × 10^6^) were incubated for 48 h in 2D or 3D culture. Total RNA was extracted from each sample and subjected to DNA microarray analysis. **a** Expression plot analysis comparing gene expression for ID8-2D and ID8-KRAS-2D cells with ID8-3D and ID8-KRAS-3D cells, respectively. **b** The number of differentially expressed genes in the microarray comparison analyses. Fold change cut-off: upregulated > 2-fold or downregulated < 2-fold

We performed cDNA microarray analysis of each cell line under different conditions (ID8-2D, ID8-3D, ID8-KRAS-2D, and ID8-KRAS-3D) to comprehensively analyze their expression profiles. All the microarray data are shown in supplementary material (Additional file 3: Supplementary file 1); Sheet 1 and Sheet 2 show ID8-2D vs. ID8-KRAS-2D and ID8-3D vs. ID8-KRAS-3D, respectively, and Sheet 3 and Sheet 4 show ID8-2D vs. ID8-3D and ID8-KRAS-2D vs. ID8-KRAS-3D, respectively). Expression plot analysis of ID8 and ID8-KRAS cells revealed similar expression pattern under 2D conditions (Fig. 2a); however, it showed significant differences under 3D conditions (Fig. 2a). The number of significantly upregulated or downregulated genes between ID8-3D and ID8-KRAS-3D cells was larger than those between ID8-2D and ID8-KRAS-2D cells (Fig. 2b). Pathway analysis revealed that genes related to cell proliferation, and cell cycle checkpoint and regulation were significantly upregulated in ID8-KRAS-3D cells than in ID8-3D cells (Fig. 2b and Additional file 4: Table S1).

### Proliferation of intraperitoneal floating cells was increased in ID8-KRAS mice

**Fig. 3:**
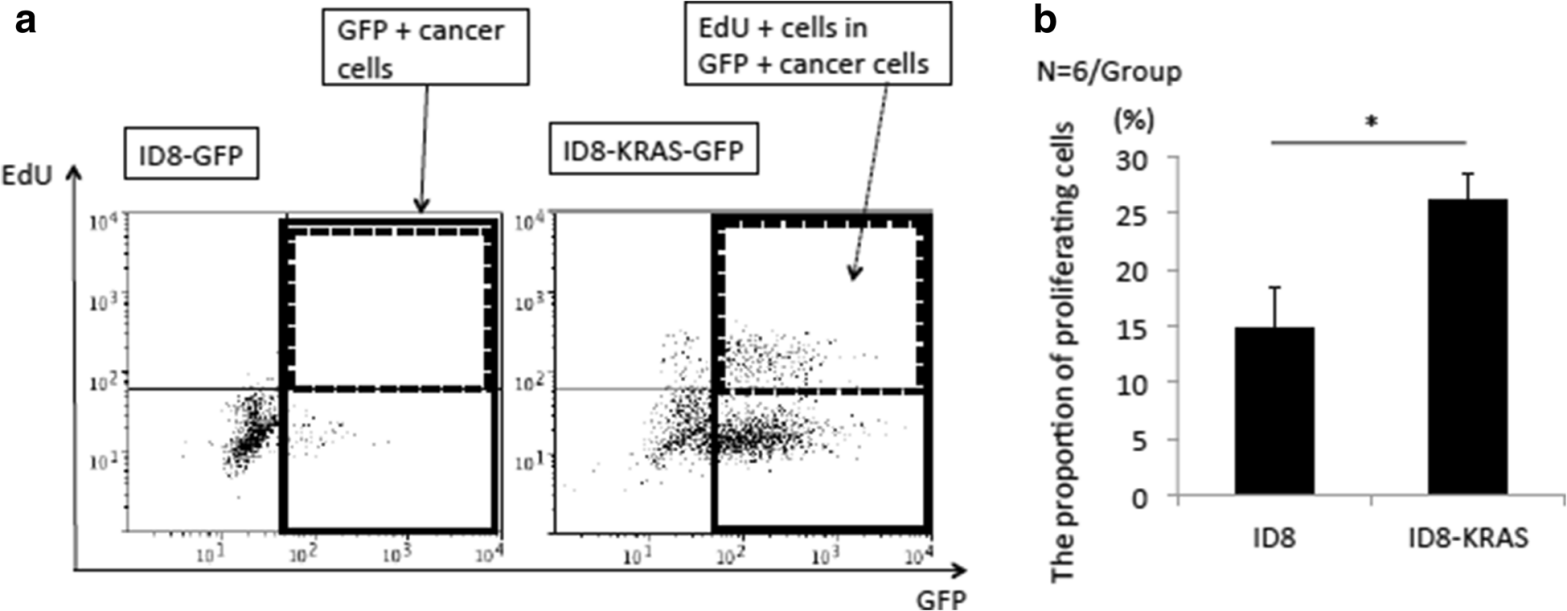
Proliferation of ID8 and ID8-KRAS cells in vivo*.* Mice were i.p. injected with EdU after 48 h of cancer cell inoculation. After 2 h of EdU administration, 8 ml of normal saline was i.p. injected into mice, and cells were recovered from the peritoneal cavity using peritoneal washes. To detect EdU incorporation, EdU was labeled with Alexa Fluor 647. **a** Comparison of the EdU uptake of ID8-GFP and ID8-KRAS-GFP cells from peritoneal washes. EdU-stained cells were analyzed by flow cytometry. The area of EdU-positive cells in GFP-positive cells was shown by the dotted frame, and that of GFP-positive cancer cells was shown by the solid frame. **b** A quantitative analysis of the EdU-positive cells in the GFP-positive cancer cells. The values shown represent the mean ± SEM (* *p* < 0.05, *n* = 6 mice per group). The proportion of proliferating cells (%) = 100 × (Number of EdU-positive cells in the cancer cell area) / (Number of all cells in the cancer cell area)

Previously, we did not observe any significant difference in the proliferation rates of disseminated cells assessed by Ki67 immunostaining between ID8 and ID8-KRAS cells [15]. In this study, we confirmed that no difference was observed in 2D culture; however, we observed obvious differences in 3D culture (Figs. 1 and 2). Therefore, we next assessed intraperitoneal floating cell proliferation by EdU uptake analysis. After intraperitoneal injection, increased number of cells in the area of lymphocytes and macrophages was observed by the FSC and SSC plot (Data not shown). The proportion of GFP-positive cancer cells obtained from peritoneal washes was 0.2 ± 0.1% for ID8 and 1.4 ± 0.6% for ID8-KRAS (*p* = 0.10) (Additional file 5: Figure S2). ID8-KRAS floating cells showed significantly higher proportion of EdU-positive cells in GFP-positive cancer cells than ID8 floating cells (ID8 14.8 ± 3.7% vs. ID8-KRAS 26.1 ± 2.4%, *p* < 0.05) (Fig. 3a and b). These results indicated that the intraperitoneal floating cancer cells in ID8-KRAS mice showed increased proliferation.

### Apoptosis induction did not differ significantly between ID8 cells and ID8-KRAS cells in 3D culture or in vivo

We next determined the apoptosis rate of each cell line in the floating condition in vitro. We assessed annexin-V staining under the four conditions (ID8-2D, ID8-3D, ID8-KRAS-2D, and ID8-KRAS-3D). There was no difference in the proportion of annexin V-positive cells between ID8-2D and ID8-KRAS-2D cells or between ID8-3D and ID8-KRAS-3D cells (ID8-2D 1.5 ± 0.3% vs. ID8-KRAS-2D 2.2 ± 0.8%, *p* = 0.46, and ID8-3D 29.3 ± 2.0% vs. ID8-KRAS-3D 27.0 ± 3.1%, *p* = 0.60) (Additional file 1: Supplementary Methods and Additional file 6: Figure S3a). We also evaluated apoptosis induction for each cell line in vivo. Because the number of floating cancer cells after 48 h of inoculation was different between ID8 and ID8-KRAS mice, we performed the apoptosis assay after 24 h of inoculation. The proportion of GFP-positive cancer cells obtained from peritoneal washes was 0.4 ± 0.2% vs. 1.8 ± 0.7%, *p* = 0.13) (Additional file 1: Supplementary Methods and Additional file 6: Figure S3b). The percentage of annexin V-stained cells among GFP-positive cancer cells did not differ between ID8 and ID8-KRAS mice (20.2 ± 4.0% vs. 17.2 ± 3.9%, *p* = 0.63) (Additional file 6: Figure S3c).

### Several molecules related to cancer progression were upregulated only in ID8-KRAS-3D cells

**Fig. 4:**
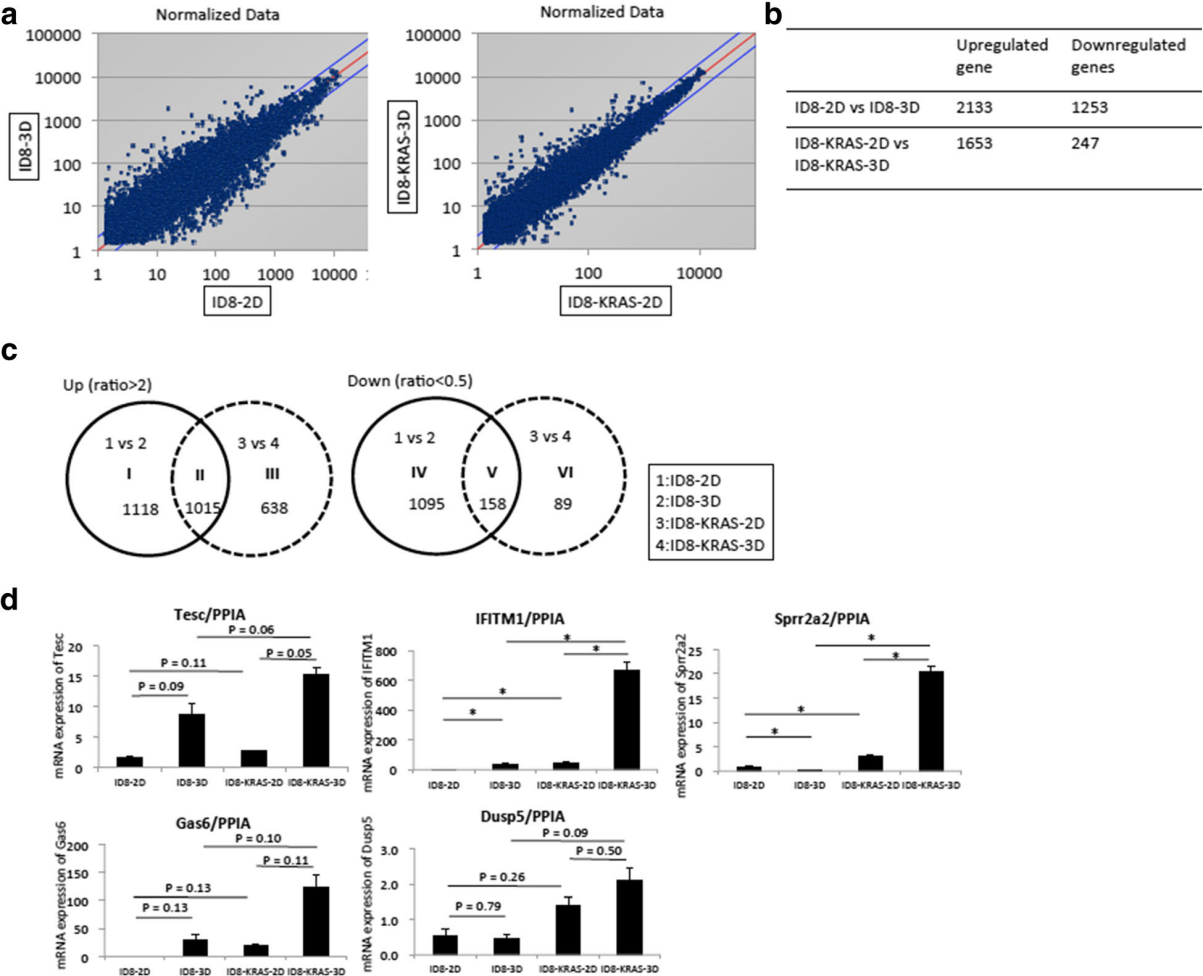
Comparison of cDNA microRNA data between ID8-2D and ID8-3D, and ID8-KRAS-2D and ID8-KRAS-3D cells, respectively. **a** Comparison of expression plot analysis of ID8-2D and ID8-3D cells with ID8-KRAS-2D and ID8-KRAS-3D cells, respectively. **b** Number of differentially expressed genes in the comparative microarray analyses. Fold change cut-off: upregulated > 2-fold or downregulated < 2-fold. **c** The Venn diagram shows the degree of intersection between modulated genes in ID8-2D and ID8-3D cells with ID8-KRAS-2D and ID8-KRAS-3D cells. **d** Expression of the genes upregulated in ID8-KRAS-3D cells. Expression mRNA levels of top 5 genes upregulated only in ID8-3D KRAS cells were evaluated by RT-qPCR. The expression of each gene level was normalized using PPIA mRNA as the internal standard. Expression levels were calculated by the comparative Ct method using PPIA as an endogenous reference gene. Error bars represent the mean ± SEM (* p < 0.05)

In an attempt to elucidate how KRAS stabilizes spheroid formation, we analyzed gene expression patterns for ID8-KRAS-3D versus ID8-KRAS-2D cells and for ID8-3D versus ID8-2D cells (Fig. 4a and b). As shown in Figs. 4, 1,015 genes were commonly upregulated in ID8-KRAS-3D and ID8-3D cells, 1118 genes were upregulated only in ID8-3D cells, and 638 genes were upregulated only in ID8-KRAS-3D cells (Fig. 4c). Since genes upregulated only in ID8-KRAS-3D cell culture might help elucidate mechanisms underlying KRAS-induced anchorage-independent proliferation, we focused our analysis on this group. Genes upregulated more than 3-fold under 3D condition than under 2D condition with normalized expression levels higher than 100 were selected as significantly altered genes (Table 1). Among these, tescalcin (Tesc), interferon-induced transmembrane protein 1 (IFITM1), growth-arrest-specific protein 6 (Gas6), and dual-specificity phosphatase 5 (DUSP5) were the most upregulated molecules. Several previous studies have shown that these genes are related to cancer progression [33–40]. Using reverse RT-qPCR, we confirmed that the expression level of these top five upregulated genes was highest in ID8-KRAS-3D cells in vitro (Fig. 4d). These results suggested that ID8-KRAS cells in 3D culture induce several molecules related to cancer progression.

**Table 1:** Genes upregulated only in ID8-KRAS-3D cell culture

Gene symbol	Global normalization			
	ID8-2D	ID8-3D	ID8-KRAS-2D	ID8-KRAS-3D
*Tesc*	286	282	347	1191
*Ifitm1*		25	132	668
*Sprr2a2*	18	14	73	550
*Gas6*		62	28	340
*Dusp5*	81	91	80	322
*Sprr2b*	14	14	63	288
*Txnip*	117	173	75	277
*Tgfbi*			4	156
*2610318N02Rik*	70	31	38	136
*Spink4*		25	2	131
*Cma1*	3	2	13	126
*Bcam*	52	93	39	124
*Vldlr*	10	12	20	120
*C1qtnf1*	30	50	25	116
*Rgs3*	25	39	29	112

The top 15 significantly altered genes were upregulated more than 3-fold compared to 2D culture and had a normalized expression level higher than 100

### MEK inhibitor suppressed cell proliferation only in 3D culture

RAS-RAF-MEK-ERK is a critical signaling pathway for KRAS-induced carcinogenesis. Furthermore, among genes upregulated only in ID8-KRAS-3D cells, *Tesc*, *Iftim1*, *Gas6,* and *Dusp5* are partially regulated by the ERK pathway [34, 41–43]. Therefore, we focused on RAS-RAF-MEK-ERK signaling as a therapeutic target. A MEK inhibitor has already been assessed in clinical trials for several cancers, including ovarian cancer [44, 45]. We used trametinib in this study as among MEK inhibitors, trametinib is approved by the Food Drug Administration and is used alone or in combination with dabrafenib to treat certain types of cancer in individuals who harbor a “*BRAF*” mutation [46].

**Fig. 5:**
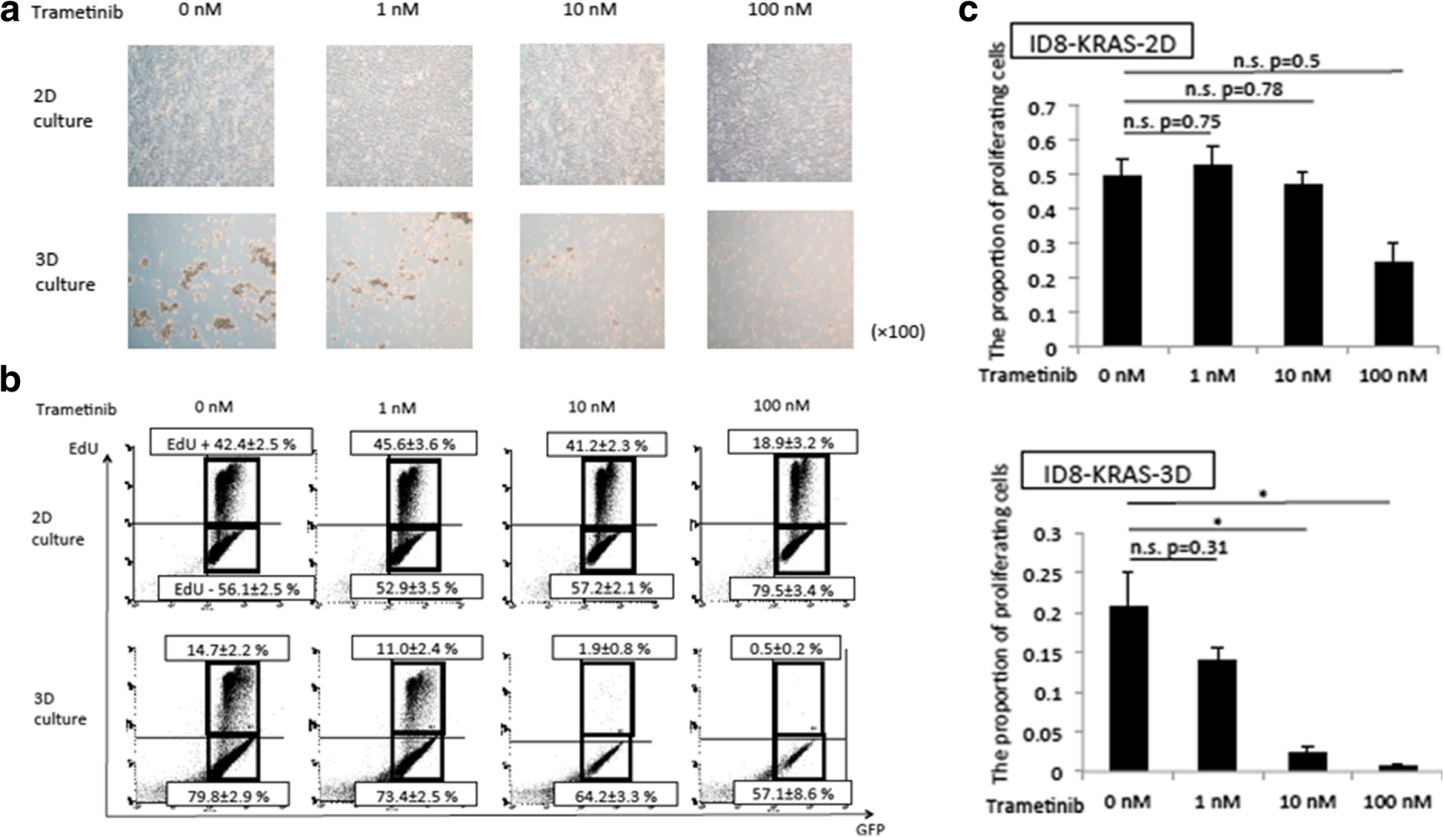
MEK inhibitor suppressed spheroid formation in ID8-KRAS cells. ID8-KRAS-GFP cells (2 × 10^5^) were not treated (0 nM) or treated with trametinib (1, 10, 100 nM) for 48 h in 2D or 3D culture. **a** Representative microscopic image of cancer cells under 2D and 3D conditions at 100× magnification. **b-c** After the treatment, cells were exposed to EdU (10 μM) for 2 h and fixed. EdU was labeled with Alexa Fluor 647. EdU uptake was analyzed (**b**) and the ratio of EdU-positive to EdU-negative cells was measured by flow cytometry (**c**). Experiments were repeated at least thrice. The values shown represent mean ± SEM (* p < 0.05)

Trametinib did not affect proliferation of cells in 2D culture, whereas it suppressed spheroid formation in a dose-dependent manner in 3D culture (Fig. 5a). >In 2D culture, trametinib did not affect the proportion of proliferation cells in any of the concentrations tested, whereas 10 nM and 100 nM trametinib decreased the proportion of proliferating cells in 3D culture (Fig. 5b and c). Thus, the MEK inhibitor trametinib suppressed spheroid formation and cell proliferation in 3D culture.

### MEK inhibitor suppressed ID8-KRAS-induced cancer formation and decreased spheroid formation in vivo

**Fig. 6:**
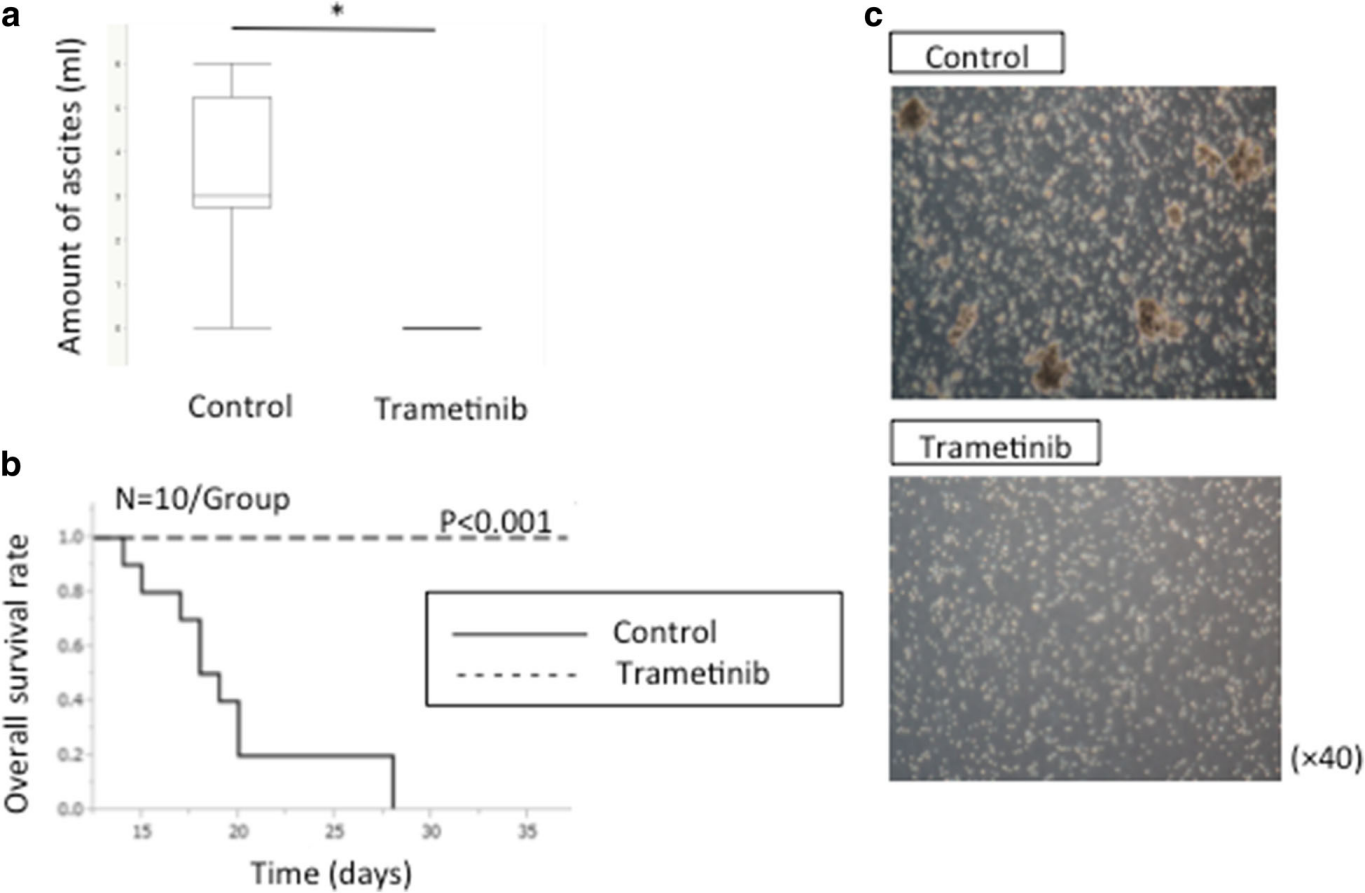
MEK inhibitor suppressed spheroid formation in ID8-KRAS mice in vivo*.* Mice were injected intraperitoneally with ID8-KRAS-GFP cells (1 × 10^6^) and treated with the MEK inhibitor trametinib or the vehicle (control). Mice were sacrificed when their body weight exceeded 23 g after inoculation. **a** Amount of ascites was assessed at the time of sacrifice. A statistical analysis was performed with the Student’s t-test (* p < 0.05). **b** Mouse survival was monitored and survival rates were quantified (*n* = 10 mice per group). Kaplan-Meier analyses using the log-rank test were performed. **c** Representative microscopic image of cancer cells in ascites or peritoneal washes collected at the time of sacrifice at 40× magnification

Finally, we assessed the efficacy of the drug treatment in vivo using ID8-KRAS mice. Trametinib treatment (1 mg/kg/day, once per day for six days a week) was started when mice were injected intraperitoneally with ID8-KRAS-GFP cells. We evaluated the volume of ascites and spheroid formation at the time of sacrifice. Trametinib markedly suppressed ascites production (control group 3.5 ± 0.5 ml vs. trametinib group 0 ml, *p* < 0.05) (Fig. 6a), and improved survival rates (*p* < 0.001) (Fig. 6b). Median survival time was 18.5 days in the control group, whereas no animals reached 23 g in the treatment group. Intraperitoneal spheroid formation was also assessed when mice were sacrificed. The spheroid formation was significantly suppressed in ID8-KRAS mice treated with trametinib (Fig. 6c). The health status of mice prior to treatment was good. MEK inhibitor treatment did not induce any adverse events. Our results suggest that trametinib suppressed spheroid formation and ascites production in peritoneal cavity and improved survival rates in ID8-KRAS mice.

## Discussion

In this study, we did not observe any significant difference between ID8 and ID8-KRAS cells in 2D culture, whereas significant differences were obvious in 3D culture and during peritoneal floating conditions in vivo. Comprehensive analysis revealed that the expression of cell cycle-related genes was significantly different between ID8 and ID8-KRAS cells in 3D culture. MEK-ERK-related factors were upregulated in ID8-KRAS-3D cells, MEK-ERK signal inhibition suppressed spheroid formation in ID8-KRAS cells, and ID8-KRAS induced cancer progression in vivo.

In our study, KRAS did not change cancer cell characteristics in 2D culture; however, it dramatically altered characteristics in 3D culture, consistent with the results of previous studies [21, 47]. ID8 cells formed few spheroid structures and could not proliferate without growth factor stimulation or interactions with TME [18]. These results suggest that ID8 cells could not stabilize spheroid formation due to lack of intracellular RAS signaling, indicating that RAS signaling might be essential for spheroid formation in ID8 cells. Studies suggest that intracellular RAS signaling promotes spheroid formation in ID8-KRAS cells, resulting in rapid tumor formation in vivo [15]. The associations of KRAS mutation with peritoneal dissemination or cancer metastases have been extensively studied. In case of colon cancer, a previous study demonstrated that patients in stage II-IV of colon cancer with mutated *KRAS* were more likely to experience disseminated disease [48]. Another study on type 1 ovarian cancer demonstrated that *KRAS* mutations are associated with poor prognosis in metastatic cancer [7].

RAS activates the RAF/MEK/ERK pathway, allowing cancer cells to proliferate in an anchorage-independent manner. Furthermore, genes upregulated only in ID8-KRAS-3D cells, such as *Tesc*, *Ifitm1*, *Gas6*, and *Dusp5,* were at least partially related to ERK pathway. For example, *Tesc* upregulation requires sustained signaling through the ERK pathway during megakaryocytic differentiation [41], and activation of ERK, PI3K, and NF-κB is required for *Ifitm1* expression [49]; Axl, the receptor of Gas6, provides a docking platform for the adaptor protein Grb2, which might be involved in the RAS-mediated ERK1/2 activation; furthermore, induction of Dusp5 is dependent on ERK1/2 activation and is involved in a negative feedback loop that inactivates the ERK1/2 MAP kinases [42]. These results suggest that RAF/MEK/ERK pathway contributes to cell proliferation under 3D conditions.

Our assessment of the therapeutic potential of a MEK-ERK inhibitor on spheroid formation revealed that the MEK inhibitor decreased spheroid formation by ID8-KRAS cells in 3D culture, but not in 2D culture. These results suggested that MEK signaling is responsible for spheroid formation in ID8-KRAS cells and it could be a therapeutic target for RAS-induced carcinogenesis. We expected trametinib to inhibit spheroid formation also in ID8 mice via inhibition of the RAS/RAF/MAPK cascade, as ID8 cells formed spheroid structures in vivo after epithelial growth factor stimulation and interactions with tumor-associated macrophages. Thus, MEK inhibitors are expected to be effective therapeutics not only for cancers with *RAS* mutations but also for cancers that depend on growth factors in the tumor microenvironment. Further investigation is needed to confirm our hypothesis.

Our results indicate that even though no obvious difference was observed under 2D conditions, cellular potential for dissemination was obvious under 3D conditions. Spheroid formation-targeting therapy has recently been an area of focus for novel cancer therapy [17]. Our results revealed that the oncogenic potential of KRAS was obvious only under 3D conditions and that spheroid formation is essential for peritoneal dissemination. Therefore, inhibition of spheroid structure formation is important for prevention of ovarian cancer dissemination. We propose that evaluation of the key signaling pathways responsible for spheroid formation could be valuable for determining the progress of dissemination. Inhibition of spheroid formation could be a therapeutic target for ovarian cancer.

We have used a mouse ovarian cancer cell line in which an obvious driver mutation has not yet been detected [23]. Human cancer cells possess various types of driver mutations; therefore, mechanisms for maintaining spheroid formation in humans might be more complicated than those revealed by this model. Additionally, as ID8 is a spontaneously transformed mouse ovarian surface epithelial (OSE) cell line, the character of ID8 is considerably different from that of human ovarian cancer. However, the OSE-derived ID8 cell line gives rise to aggressive, widely disseminated cancers that are pathologically and histologically similar to human HGSC [23, 50].

In this study, we demonstrated that KRAS promotes ovarian cancer cell dissemination by stabilizing spheroid formation and that the MEK pathway is responsible for stabilized spheroid formation. An increasing number of studies on ovarian cancer are using spheroid cultures to recapitulate the mechanisms that promote neoplastic transformation and metastasis by creating a 3D environment in vitro that mimics human tissue [51]. Our study has added new insights into this growing field.

## Conclusions

We showed that KRAS promotes ovarian cancer cell dissemination by stabilizing spheroid formation and that the MEK pathway is responsible for stabilized spheroid formation. Therefore, suppression of spheroid formation could be a therapeutic target for cancer cell dissemination.

## Additional files

Additional file 1: Supplementary methods. Methods of western blot analysis, in vitro apoptosis assessment using Annexin-V and in vivo apoptosis assessment. (DOCX 20 kb)

Additional file 2: **Figure S1.** KRAS activation by evaluating the downstream protein of RAS cascade. ID8-GFP and ID8-KRAS-GFP cells (1 × 10^6^) were incubated for 48 hours. Attached cells were collected, washed with PBS, and proteins were extracted from cells. Cell lysates were subjected to Western blotting analysis for expression of phospho-MEK1/2, MEK1/2, phospho-ERK1/2, ERK1/2 and anti-β-actin. (PDF 68 kb)

Additional file 3: Supplementary File 1. All the cDNA microarray data for analysis of ID8-2D, ID8-3D, ID8-KRAS-2D, and ID8-KRAS-3D. Sheet 1 and Sheet 2 show ID8-2D vs. ID8-KRAS-2D and ID8-3D vs. ID8-KRAS-3D, respectively, and Sheet 3 and Sheet 4 show ID8-2D vs. ID8-3D and ID8-KRAS-2D vs. ID8-KRAS-3D, respectively. (XLSX 900 kb)

Additional file 4: **Table S1.** KEGG pathway analysis for genes upregulated in ID8-KRAS-3D cells compared to ID8-3D cells. (DOCX 85 kb)

Additional file 5: **Figure S2.** GFP-positive cancer cells in ID8 and ID8-KRAS cells in vivo. Mice were i.p. injected with EdU after 48 h of cancer cell inoculation. After 2 h of EdU administration, 8 ml of normal saline was i.p. injected into mice, and cells were recovered from the peritoneal cavity using peritoneal washes. EdU-stained cells were analyzed by flow cytometry. A quantitative analysis of the GFP-positive cancer cells in total cells obtained from peritoneal washes. The values shown represent the mean ± SEM (* p < 0.05, *n* = 6 mice per group). (PDF 12 kb)

Additional file 6: **Figure S3.** Assessment of apoptosis in ID8 and ID8-KRAS cells in vitro and in vivo. a ID8 and ID8-KRAS cells (1 × 10^6^) were incubated for 48 hours in 2D or 3D culture. Floating and attached cells were collected, washed with PBS, and subjected to PI/Annexin-V staining. Annexin V-FITC (5 μl) and propidium iodide (5 μl, 50 μg/ml) were added to the cell suspension. The stained cells were analyzed and the percentage of PI-negative/Annexin-V-positive apoptotic cells was measured by flow cytometry. Experiments were repeated at least three times. The values shown represent the mean ± SEM (*p < 0.05). b,c Mice were i.p. injected with ID8-GFP or ID8-KRAS-GFP cells (1 × 10^6^). Peritoneal washes were collected 24 hours later. ID8-GFP and ID8-KRAS-GFP cells were collected by centrifugation, washed with PBS, and subjected to Annexin-V staining. The stained cells were analyzed by flow cytometry. A quantitative analysis of the percentage of the GFP-positive cancer cells in total cells obtained from peritoneal washes (b) and the percentage of apoptotic cells in GFP-positive cancer cells (c). The values shown represent the mean ± SEM (*p < 0.05, *n* = 6 mice per group). (PDF 29 kb)

## Abbreviations

BW: Body weight
DMEM: Dulbecco’s modified Eagle’s medium
DMSO: Dimethyl sulfoxide
DUSP5: Dual-specificity phosphatase 5
EdU: 5-ethnyl-2′-deoxyuridine
FBS: Fetal bovine serum
FDR: False discovery rate
FSC: Forward scatter
Gas6: Growth-arrest-specific protein 6
HGSC: High-grade serous carcinoma
IFITM1: Interferon-induced transmembrane protein 1
KEGG: Kyoto Encyclopedia of Genes and Genomes
LGSC: Low-grade serous carcinoma
NSCLC: Non-small cell lung cancer
PBS: Phosphate buffered saline
PPIA: Peptidylprolyl isomerase A
RT-qPCR: Reverse transcription-quantitative polymerase chain reaction
SEM: Standard error of mean
Sprr2a2: Small proline-rich protein 2A2
SSC: Side scatter
TCGA: The Cancer Genome Atlas
Tesc: Tescalcin
TME: Tumor microenvironment
