# Supplementary material for: The oncogene KRAS promotes cancer cell dissemination by stabilizing spheroid formation via the MEK pathway
Source: BMC Cancer. 2018 Dec 3;18:1201. doi: 10.1186/s12885-018-4922-4 (PMC6278087; doi:10.1186/s12885-018-4922-4)
Supplement: Supplementary file 1 — Supplementary methods. Methods of western blot analysis, in vitro apoptosis assessment using Annexin-V and in vivo apoptosis assessment. (DOCX 20 kb) [file 12885_2018_4922_MOESM1_ESM.docx]

**Additional file 1:**

**Supplementary Methods**

**Western blot analysis**

ID8-GFP and ID8-KRAS-GFP cells (1 × 10^6^) were cultured in serum-containing media for 48 hours. Proteins were extracted from cells in Radio-immunoprecipitation assay buffer (Wako) containing cOmplete, Mini protease inhibitor cocktail (Sigma-Aldrich, MO, USA) on ice for 10 min. Lysates were then sonicated briefly and centrifuged at 14,000 rpm at 4 ℃ for 15 min. The supernatants were analyzed. Protein concentration was measured by Protein assay dye reagent concentrate (Bio-Rad, CA, USA). Equivalent amounts of lysate protein (10 μg) were subjected to Mini-PROTEAN TGX Precast Protein Gels (Bio-Rad) and separated by SDS-PAGE. And they were electrophoretically transferred onto Trans-Blot Turbo Transfer Packs (Bio-Rad) using Trans-Blot Turbo Transfer System (Bio-Rad), incubated with specific primary antibodies, incubated with appropriate secondary antibodies, and analyzed with the Immobilon Western Chemiluminescent HRP substrate (Merck Millipore) according to the manufacturer's instructions. Antibodies used included those directed against phospho-MEK1/2, MEK1/2, phospho-ERK1/2, ERK1/2 (Cell Signaling Technology, Beverly, MA) and anti-β-actin (Sigma-Aldrich). Peroxidase-based chemiluminescence was developed by addition of ECL, and the bands were detected using an LAS 3000 image analyzer (Fuji Film, Tokyo, Japan).

***In vitro* Apoptosis assessment using Annexin-V**

Apoptosis was measured with flow cytometry using an Annexin-V-FITC Apoptosis Detection Kit (Abcam, MA, USA). ID8 and ID8-KRAS cells (1 × 10^6^) were cultured in serum-containing media for 48 hours in 2D or 3D culture. Floating and attached cells were both collected, washed with PBS, and resuspended in buffer containing Annexin-V and PI according to the manufacturer’s instructions. The proportions of Annexin-V-positive cells were examined. All samples were acquired using a FACSCalibur system and analyzed using FlowLogic software (Inivai Technologies).

***In vivo* apoptosis assessment**

For *in vivo* analysis of apoptosis, mice were i.p. injected with ID8-GFP or ID8-KRAS-GFP cells (1 × 10^6^) on first day. On second day after 24 h of injection of ID8-GFP or ID8-KRAS-GFP cells, 8 ml of normal saline were i.p. injected into mice, recovered from peritoneal cavity as peritoneal washes and passed through a cell strainer. About 1,000,000 cells were obtained routinely from recovered peritoneal washes per mouse. The peritoneal washes were centrifuged for 5 min at 1500 rpm and the supernatant was aspirated, and red blood cells were lysed. To detect apoptosis, cell suspension in binding buffer was labeled with Annexin-V-EnzoGold (enhanced Cyanine-3) using the GFP-certified Apoptosis/Necrosis detection kit (Enzo, NY, USA) according to the manufacturer’s protocol. Stained cells were run on a FACSCalibur flow cytometer (BD Biosciences, NJ, USA). The cancer cell area was recognized by the Forward Scatter (FSC) versus Side Scatter (SSC) plot and GFP fluorescence. The number of apoptotic cells was normalized by the number of cancer cells gated in the GFP-positive area. The data were analyzed using FlowLogic software (Inivai Technologies, Melbourne, Australia).
