# Supplementary figures and images for: The oncogene KRAS promotes cancer cell dissemination by stabilizing spheroid formation via the MEK pathway
Source: BMC Cancer. 2018 Dec 3;18:1201. doi: 10.1186/s12885-018-4922-4 (PMC6278087; doi:10.1186/s12885-018-4922-4)

Fig. S1

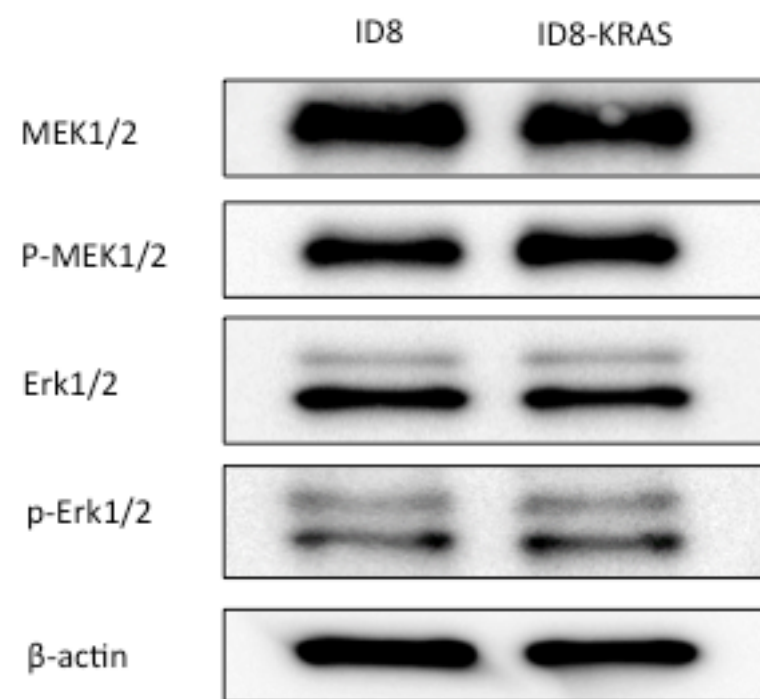

Supplement: Supplementary file 2 — Figure S1. KRAS activation by evaluating the downstream protein of RAS cascade. ID8-GFP and ID8-KRAS-GFP cells (1 × 10^6^) were incubated for 48 hours. Attached cells were collected, washed with PBS, and proteins were extracted from cells. Cell lysates were subjected to Western blotting analysis for expression of phospho-MEK1/2, MEK1/2, phospho-ERK1/2, ERK1/2 and anti-β-actin. (PDF 68 kb) [file 12885_2018_4922_MOESM2_ESM.pdf]

Fig. S2

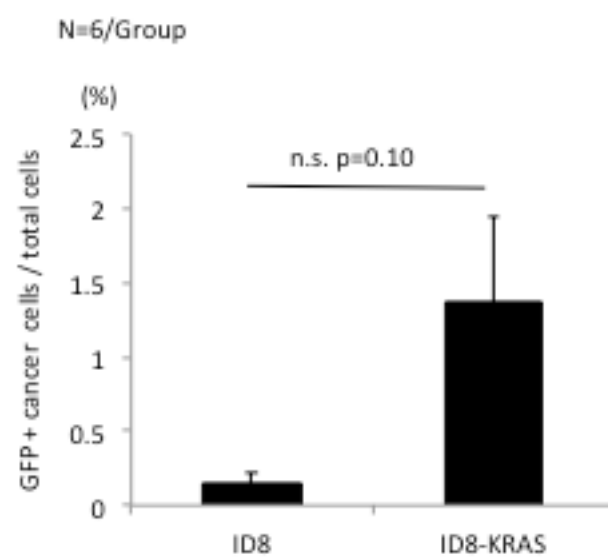

Supplement: Supplementary file 5 — Figure S2. GFP-positive cancer cells in ID8 and ID8-KRAS cells in vivo. Mice were i.p. injected with EdU after 48 h of cancer cell inoculation. After 2 h of EdU administration, 8 ml of normal saline was i.p. injected into mice, and cells were recovered from the peritoneal cavity using peritoneal washes. EdU-stained cells were analyzed by flow cytometry. A quantitative analysis of the GFP-positive cancer cells in total cells obtained from peritoneal washes. The values shown represent the mean ± SEM (* p < 0.05, n = 6 mice per group). (PDF 12 kb) [file 12885_2018_4922_MOESM5_ESM.pdf]

Fig. S3

a

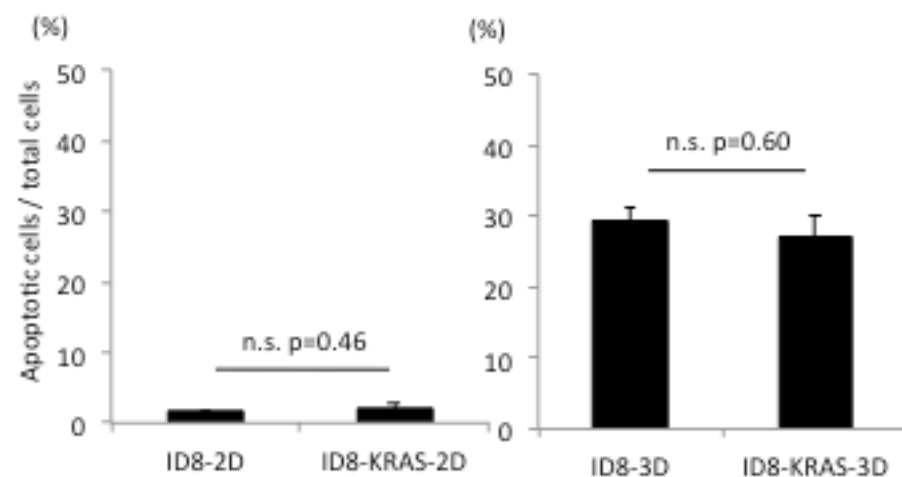

b

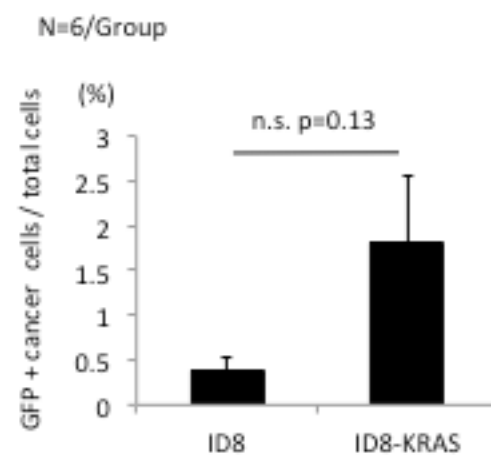

c

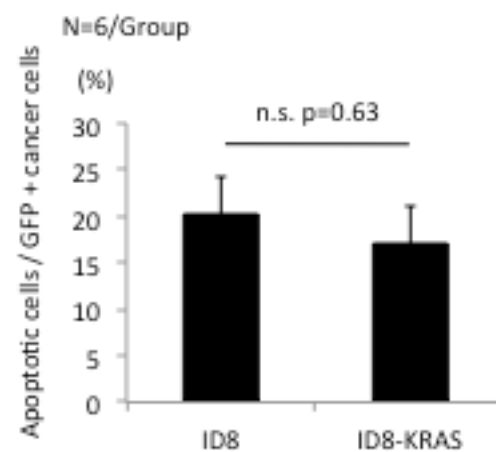

Supplement: Supplementary file 6 — Figure S3. Assessment of apoptosis in ID8 and ID8-KRAS cells in vitro and in vivo. a ID8 and ID8-KRAS cells (1 × 10^6^) were incubated for 48 hours in 2D or 3D culture. Floating and attached cells were collected, washed with PBS, and subjected to PI/Annexin-V staining. Annexin V-FITC (5 μl) and propidium iodide (5 μl, 50 μg/ml) were added to the cell suspension. The stained cells were analyzed and the percentage of PI-negative/Annexin-V-positive apoptotic cells was measured by flow cytometry. Experiments were repeated at least three times. The values shown represent the mean ± SEM (*p < 0.05). b,c Mice were i.p. injected with ID8-GFP or ID8-KRAS-GFP cells (1 × 10^6^). Peritoneal washes were collected 24 hours later. ID8-GFP and ID8-KRAS-GFP cells were collected by centrifugation, washed with PBS, and subjected to Annexin-V staining. The stained cells were analyzed by flow cytometry. A quantitative analysis of the percentage of the GFP-positive cancer cells in total cells obtained from peritoneal washes (b) and the percentage of apoptotic cells in GFP-positive cancer cells (c). The values shown represent the mean ± SEM (*p < 0.05, n = 6 mice per group). (PDF 29 kb) [file 12885_2018_4922_MOESM6_ESM.pdf]
