# Supplementary material for: The oncogene KRAS promotes cancer cell dissemination by stabilizing spheroid formation via the MEK pathway
Source: BMC Cancer. 2018 Dec 3;18:1201. doi: 10.1186/s12885-018-4922-4 (PMC6278087; doi:10.1186/s12885-018-4922-4)
Supplement: Supplementary file 4 — Table S1. KEGG pathway analysis for genes upregulated in ID8-KRAS-3D cells compared to ID8-3D cells. (DOCX 85 kb) [file 12885_2018_4922_MOESM4_ESM.docx]

**Additional file 4:**

**Supplementary Tabl**e **1.** **KEGG pathway analysis for genes upregulated in ID8-KRAS-3D cells compared to ID8-3D cells**

| Pathway annotation | Genes | NG^a^ | p*^b^ |
| --- | --- | --- | --- |
| (KEGG) 04110: Cell cycle | Ccnb1, Mad2l1, Cdk1, Orc1, Chek2, Mcm3, Ccna2, Pcna, Espl1, Ccnd1, Bub1, Skp2, Dbf4, Orc6, Rbl1, Mcm4, Chek1, E2f1, Mcm6, Cdkn2d, Cdc25b, Cdc20, Mcm2, Bub1b, Cdc6, Wee1, Ccnb2, Ccne1, Cdc45, Anapc7, Cdc25c, Plk1, Mcm7, Mcm5, Ttk | 35 | 4.97E-30 |
| (KEGG) 03030: DNA replication | Mcm3, Pcna, Pola1, Prim2, Pole, Rfc5, Prim1, Mcm4, Rfc2, Lig1, Mcm6, Pola2, Mcm2, Pold2, Rnaseh2b, Rfc3, Rpa2, Mcm7, Fen1, Mcm5, Pole2, Rpa3 | 22 | 1.81E-28 |
| (KEGG) 00240: Pyrimidine metabolism | Tk1, Pola1, Ctps, Prim2, Pole, Dut, Cda, Umps, Prim1, Dck, Upp1, Pola2, Pold2, Dtymk, Dctd, Tyms, Rrm2, Polr3g, Pole2, Rrm1, Uck2 | 21 | 1.31E-15 |
| (KEGG) 03420: Nucleotide excision repair  (KEGG) 03030: DNA replication | Pcna, Pole, Rfc5, Rfc2, Lig1, Pold2, Rfc3, Rpa2, Pole2, Rpa3 | 10 | 3.59E-12 |
| (KEGG) 03410: Base excision repair | Gm5518, Pcna, Pole, Lig1, Neil3, Pold2, Hmgb1, Tdg, Fen1, Pole2, Mutyh, Ung | 12 | 8.87E-12 |
| (KEGG) 04110: Cell cycle  (KEGG) 03030: DNA replication | Mcm3, Pcna, Mcm4, Mcm6, Mcm2, Mcm7, Mcm5 | 7 | 1.13E-11 |
| (KEGG) 03420: Nucleotide excision repair  (KEGG) 03030: DNA replication  (KEGG) 03430: Mismatch repair | Pcna, Rfc5, Rfc2, Lig1, Pold2, Rfc3, Rpa2, Rpa3 | 8 | 4.67E-10 |
| (KEGG) 04110: Cell cycle  (KEGG) 04914: Progesterone-mediated oocyte maturation | Ccnb1, Mad2l1, Cdk1, Ccna2, Bub1, Cdc25b, Ccnb2, Anapc7, Cdc25c, Plk1 | 10 | 6.88E-10 |
| (KEGG) 03430: Mismatch repair | Pcna, Rfc5, Rfc2, Exo1, Lig1, Pold2, Rfc3, Rpa2, Rpa3 | 9 | 9.50E-10 |
| (KEGG) 04114: Oocyte meiosis | Mad2l1, Cdk1, Espl1, Bub1, Aurka, Ppp2r1b, Sgol1, Calml3, Rps6ka2, Cdc20, Ccnb2, Ccne1, Anapc7, Cdc25c, Plk1, Fbxo5 | 16 | 1.05E-09 |

^a^ NG: Number of genes in the input list with the given annotations

^b^ p*: p-value corrected for multiple hypothesis testing or false discovery rate (FDR) method
